# The ERACE-PA Global Surveillance Program: Ceftolozane/tazobactam and Ceftazidime/avibactam in vitro Activity against a Global Collection of Carbapenem-resistant *Pseudomonas aeruginosa*

**DOI:** 10.1007/s10096-021-04308-0

**Published:** 2021-07-22

**Authors:** Christian M. Gill, Elif Aktaþ, Wadha Alfouzan, Lori Bourassa, Adrian Brink, Carey-Ann D. Burnham, Rafael Canton, Yehuda Carmeli, Marco Falcone, Carlos Kiffer, Anna Marchese, Octavio Martinez, Spyros Pournaras, Michael Satlin, Harald Seifert, Abrar K. Thabit, Kenneth S. Thomson, Maria Virginia Villegas, David P. Nicolau, the ERACE-PA Global Study Group

**Affiliations:** 1 Center for Anti-Infective Research & Development Hartford Hospital, 80 Seymour Street, Hartford, CT 06102 USA; 2 Clinical Microbiology Laboratory, University of Health Sciences, Sisli Hamidiye Etfal Training and Research Hospital, Istanbul, Turkey; 3 Laboratory Medicine- Farwania Hospital, Ministry of Health, Kuwait, Department of Microbiology, Faculty of Medicine, Kuwait University, Jabriya, Kuwait; 4 Department of Laboratory Medicine and Pathology, University of Washington, Seattle, WA USA; 5 Division of Medical Microbiology, Department of Pathology, Faculty of Health Sciences, National Health Laboratory Services, University of Cape Town, Cape Town , South Africa; 6 Washington University in St. Louis School of Medicine, St. Louis, MO USA; 7 Servicio de Microbiologia. Hospital Ramón Y Cajal-IRYCIS, Madrid, Spain; 8 National Institute for Infection Control and Antibiotic Resistance, Tel-Aviv Sourasky Medical Center, Tel-Aviv, Israel; 9 Infectious Diseases Division, Department of Clinical and Experimental Medicine, University of Pisa, Pisa, Italy; 10 Internal Medicine Department and LEMC-Alerta Lab, Escola Paulista de Medicina, UNIFESP, São Paulo, Brazil; 11 Department of Surgical Sciences and Integrated Diagnostics (DISC), University of Genoa, and Clinical Microbiology Unit, San Martino Policlinico Hospital—IRCCS for Oncology and Neuroscience, Genoa, Italy; 12 Department of Pathology and Microbiology, University of Miami Miller School of Medicine, Miami, FL USA; 13 Laboratory of Clinical Microbiology, Attikon University Hospital, School of Medicine, National and Kapodistrian University of Athens, Athens, Greece; 14 Division of Infectious Diseases, Department of Medicine, Weill Cornell Medicine, New York, NY USA; 15 Institute for Medical Microbiology, Immunology and Hygiene, University of Cologne, Goldenfelsstrasse 19-21, 50935 Köln, Germany; 16 Pharmacy Practice Department, Faculty of Pharmacy, King Abdulaziz University, Jeddah, Saudi Arabia; 17 University of Louisville School of Medicine, Louisville, KY USA; 18 Grupo de Resistencia Antimicrobiana Y Epidemiología Hospitalaria (RAEH), Universidad El Bosque, Bogotá, Colombia; 19 Division of Infectious Diseases, Hartford Hospital, Hartford, CT USA

**Keywords:** Carbapenem-resistant *P. aeruginosa*, Ceftazidime/avibactam, Ceftolozane/tazobactam, Carbapenemase

## Abstract

The cephalosporin-β-lactamase-inhibitor-combinations, ceftolozane/tazobactam and ceftazidime/avibactam, have revolutionized treatment of carbapenem-resistant *Pseudomonas aeruginosa* (CR-PA). A contemporary assessment of their in vitro potency against a global CR-PA collection and an assessment of carbapenemase diversity are warranted. Isolates determined as CR-PA by the submitting site were collected from 2019–2021 (17 centers in 12 countries) during the ERACE-PA Global Surveillance Program. Broth microdilution MICs were assessed per CLSI standards for ceftolozane/tazobactam, ceftazidime/avibactam, ceftazidime, and cefepime. Phenotypic carbapenemase testing was conducted (modified carbapenem inactivation method (mCIM)). mCIM positive isolates underwent genotypic carbapenemase testing using the CarbaR, the CarbaR NxG, or whole genome sequencing. The MIC_50/90_ was reported as well as percent susceptible (CLSI and EUCAST interpretation). Of the 807 isolates, 265 (33%) tested carbapenemase-positive phenotypically. Of these, 228 (86%) were genotypically positive for a carbapenemase with the most common being VIM followed by GES. In the entire cohort of CR-PA, ceftolozane/tazobactam and ceftazidime/avibactam had MIC_50/90_ values of 2/ > 64 and 4/64 mg/L, respectively. Ceftazidime/avibactam was the most active agent with 72% susceptibility per CLSI compared with 63% for ceftolozane/tazobactam. For comparison, 46% of CR-PA were susceptible to ceftazidime and cefepime. Against carbapenemase-negative isolates, 88 and 91% of isolates were susceptible to ceftolozane/tazobactam and ceftazidime/avibactam, respectively. Ceftolozane/tazobactam and ceftazidime/avibactam remained highly active against carbapenem-resistant *P. aeruginosa*, particularly in the absence of carbapenemases. The contemporary ERACE-PA Global Program cohort with 33% carbapenemase positivity including diverse enzymology will be useful to assess therapeutic options in these clinically challenging organisms with limited therapies.

## Introduction

Multi-drug resistant *Pseudomonas aeruginosa* burdens clinicians across the globe due to the limited treatment options [1]. *P. aeruginosa* represents such a challenging pathogen due to the numerous mechanisms that drive antimicrobial resistance including drug efflux/porin loss, endogenous/exogenous β-lactamases, and target site mutations [2]. Although resistance mechanisms and epidemiology may differ based on geographic region, resistance to carbapenems is noted around the globe leaving clinicians agents that may be less effective and/or more toxic than β-lactams (i.e., polymyxins, aminoglycosides) [1]. Between 2014 and 2015, novel cephalosporin-β-lactamase-inhibitor combinations, ceftolozane/tazobactam and ceftazidime/avibactam, were introduced and revolutionized the treatment of carbapenem-resistant *P. aeruginosa* [3, 4].

Since introduction, both ceftolozane/tazobactam and ceftazidime/avibactam have shown potent activity against clinical *P. aeruginosa* isolates including carbapenem-resistant isolates [5]. The potent in vitro activity translated to improved patient outcomes compared to best available therapies by improving efficacy and safety [6–8]. However, now years into both agents representing important therapies for susceptible carbapenem-resistant *P. aeruginosa* where other β-lactams are ineffective, resistance has been described. Plasmid-mediated resistance due to carbapenemase production, including metallo-β-lactamases, has been a noted clinical challenge since introduction of both therapies due to β-lactam cross-resistance and global spread of such organisms increases concerns [9]. Similarly, mutations to chromosomally encoded *P. aeruginosa* derived cephalosporinases (PDCs) and transmissible extended-spectrum β-lactamases have been described also resulting in ceftolozane/tazobactam and ceftazidime/avibactam resistance [10, 11]. Indeed, a regional assessment from a global program of the in vitro activity of these agents 5 years later against the targeted pathogen of carbapenem-resistant *P. aeruginosa* will help clinicians assess the activity of these agents in their region.

Herein, we describe the establishment of the Enhancing Rational Antimicrobials against Carbapenem-resistant *P. aeruginosa* (ERACE-PA) Global Surveillance Program. This is a multi-center, multi-national surveillance program comprised of carbapenem-resistant *P. aeruginosa* submitted from around the globe. The program represents a contemporary assessment of the in vitro potency of ceftolozane/tazobactam and ceftazidime/avibactam 5 years into use. Additionally, the carbapenemase diversity of included isolates was assessed to categorize the cohort.

## Methods

### Bacterial isolates

Isolates were compiled as part of the ERACE-PA Global Surveillance Program. A total of 17 sites from 12 countries were included in the program. Global sites were located in Köln, Germany; Sao Paulo, Brazil; Istanbul, Turkey; Tel Aviv, Israel; Madrid, Spain; Jabriya, Kuwait; Cape Town, South Africa; Bogotá, Colombia; Athens, Greece; Jeddah, Saudi Arabia; Pisa, Italy; and Genoa, Italy. In the USA, centers from New York, NY; Miami, FL; St. Louis, MO; Seattle, WA; and Louisville, KY, submitted isolates. Isolates were sent to the central laboratory (Center for Anti-Infective Research and Development, Hartford, CT) for storage frozen at − 80 ^o^ C in skim milk until assessment.

Isolates could be included if they were non-duplicate isolates identified as *P. aeruginosa* by local standards of practice and determined to be carbapenem-resistant by the submitting site. Isolates were collected from 2019 to 2021. Isolates could be cultured from any anatomical site and there was no patient age limit for inclusion.

### In vitro* susceptibility testing*

Isolates were transferred from frozen stock and then subsequently subcultured once more prior to all testing. Reference broth microdilution MICs were conducted at the central laboratory per CLSI standards to ceftolozane/tazobactam, ceftazidime/avibactam, ceftazidime, and cefepime [12, 13]. Routine quality control was conducted after tray preparation and during each MIC run using either ATCC *P. aeruginosa* ATCC 27853 or ATCC *K. pneumoniae* 700603. MICs were read after 16–20 h incubation and colony counts were conducted for each inoculum to confirm the target bacterial burden was transferred to the MIC trays by transferring one µL from a control well onto a trypticase soy agar with 5% sheep’s blood plate which was subsequently counted after overnight incubation.

### Phenotypic carbapenemase screening

All isolates underwent phenotypic carbapenemase testing at the central laboratory using the modified carbapenem inactivation method (mCIM) per CLSI standards and interpreted by CLSI standards [12]. Routine quality control was conducted with each mCIM run with two negative controls (*P. aeruginosa* ATCC 27,853 and ATCC BAA *K. pneumoniae* 1706) and two positive controls (ATCC BAA *K. pneumoniae* 1705 (KPC-positive) and *K. pneumoniae* CDC #766 (NDM-positive).

### Genotypic carbapenemase detection

Any isolates that tested mCIM positive were then assessed on the CarbaR assay (Cepheid, Sunnyvale, CA, USA) per the manufacturer’s package insert. Results were determined as positive for KPC, NDM, VIM, IMP, OXA-48-like, or negative for all targets.

All isolates that tested negative on the commercially available CarbaR were sent to Cepheid for assessment on the Research Use Only CarbaR NxG as previously described [14]. NxG testing assessed for the presence of more carbapenemase targets including GES, SPM, IMI, OXA-58, and IMP-subtypes.

Isolates negative for both assays underwent whole genome sequencing as previously described to evaluate for enzymatic resistance mechanisms outside of the CarbaR and CarbaR NxG spectrum [14].

Additional CarbaR NxG testing was conducted on ceftolozane/tazobactam-resistant isolates that tested mCIM negative to evaluate for GES-harboring isolates as this enzymology has previously been described as testing falsely negative [15, 16].

### Clinical data

The present study was approved by the Hartford Hospital institutional review board and determined as exempted as all patient care was delivered per standards of care in the past, and thus, written informed consent was not obtained. De-identified clinical data of sex, age, hospital level of care at time of culture (intensive care unit (ICU), ward, or outpatient), and source of infection (respiratory, blood, urine, intra-abdominal, or other) were collected. Pediatric patients were defined as patients age < 18 years old.

### Analysis

The categorical interpretation of the MIC for each agent was determined using CLSI and EUCAST interpretive criteria and described as percent susceptible, intermediate, and resistant (as applicable) in the entire cohort and subgroups [12, 17]. Demographic data was assessed using descriptive statistics including percentages for categorical data. For continuous data, the mean and standard deviation was reported.

## Results

### Demographics

A total of 807 isolates were collected. The mean age of patients was 56 (± 21) years-old and 62% of patients were male. A total of 46 isolates (7%) were obtained from patients less than 18 years old. The majority of patients were on inpatient wards (54%) at the time of culture, 37% were ICU patients. The respiratory tract represented the most common identified source (41%) followed by urine (20%) and blood (11%). Full demographic data are presented in Table 1.

**Table 1:** Demographic data for the patients corresponding to submitted isolates

Demographic data	Mean (SD) or n (%)
Age (years), mean (SD)	56 (± 21)
Sex, Percent male	62%
Location at time of culture, percent of isolates	
Ward	54%
ICU	37%
Outpatient	2%
Unspecified	7%
Source	
Respiratory	41%
Urine	20%
Blood	11%
Intra-abdominal	2%
Other	26%
Region, n (%)	
Europe	324 (40%)
Middle East	163 (20%)
USA	149 (19%)
South America	106 (13%)
Africa	65 (8%)

### Carbapenemase assessment

Phenotypic detection of a carbapenemase was noted for 265 of the 807 (33%) isolates. A total of 228 of the 265 (86%) phenotypically positive isolates had a carbapenemase gene detected by molecular testing (Table 2). Carbapenemase prevalence varied by region with the highest prevalence rates in Africa and Middle East with 68 and 46% of isolates from each region, respectively.

**Table 2:** Carbapenemase diversity of the entire cohort and by region

Cohort Subgroups, Number (Percent of each Subgroup)	Number (% of carbapenemase positive)
Entire Cohort, n = 280 (35%)	
VIM	136 (49%)
GES	59 (21%)
IMP	15 (5%)
NDM	13 (5%)
KPC	8 (3%)
VIM and KPC	8 (3%)
VIM and IMP	3 (1%)
VIM and OXA-48	1 (< 1%)
Other non-carbapenemase β-lactamases	37 (13%)
Europe, *n* = 109 (35%)	
VIM	48 (44%)
GES	40 (37%)
NDM	1 (1%)
Other non-carbapenemase β-lactamases	20 (18%)^a^
Middle East, *n* = 75 (46%)	
VIM	28 (37%)
GES	18 (24%)
IMP	13 (17%)
NDM	8 (11%)
VIM and IMP	3 (4%)
Other non-carbapenemase β-lactamases	5 (7%)^b^
USA, *n* = 17 (11%)	
VIM	10 (59%)
Other non-carbapenemase β-lactamase	7 (41%)^c^
South America, *n* = 35 (33%)	
VIM	15 (42%)
IMP	2 (6%)
KPC	8 (23%)
VIM and KPC	8 (23%)
Other non-carbapenemase β-lactamases	2 (6%)^d^
Africa, *n* = 44 (68%)	
VIM	35 (80%)
GES	1 (2%)
NDM	4 (9%)
VIM and OXA-48	1 (2%)
Other non-carbapenemase β-lactamases	3 (7%)^e^

^a^OXA-50-like + PDC, n = 1; OXA-10-like + OXA-50-like + PDC, n = 2; not sequenced but from same site and similar phenotype as the OXA-10-like + OXA-50-like + PDC isolates, n = 11, WGS unavailable, n = 6

^b^OXA-50-like + PDC, n = 3; OXA-2-like + OXA-50-like + PDC, n = 2

^c^OXA-50-like + PDC, n = 3; OXA-2 + OXA-50-like + PDC, n = 1; not sequenced but from same site and similar phenotype to OXA-50-like + PDC isolates, n = 2, WGS unavailable, n = 1

^d^OXA-2 + OXA-50-like + PDC, n = 1; OXA-50-like + PDC, n = 1

^e^OXA-50-like + PDC, n = 1; OXA-10-like + OXA-50-like + PDC, n = 2

The most common carbapenemase genotypically identified was VIM (49%) followed by GES (21%). A total of 15 genotypically GES-categorized isolates tested mCIM-negative. The diversity of carbapenemase enzymology is presented in Table 2. Twelve isolates co-harbored two carbapenemase genes including nine harboring both metallo- and serine-carbapenemases.

### Ceftolozane/tazobactam and ceftazidime/avibactam in vitro activity

**Fig. 1:**
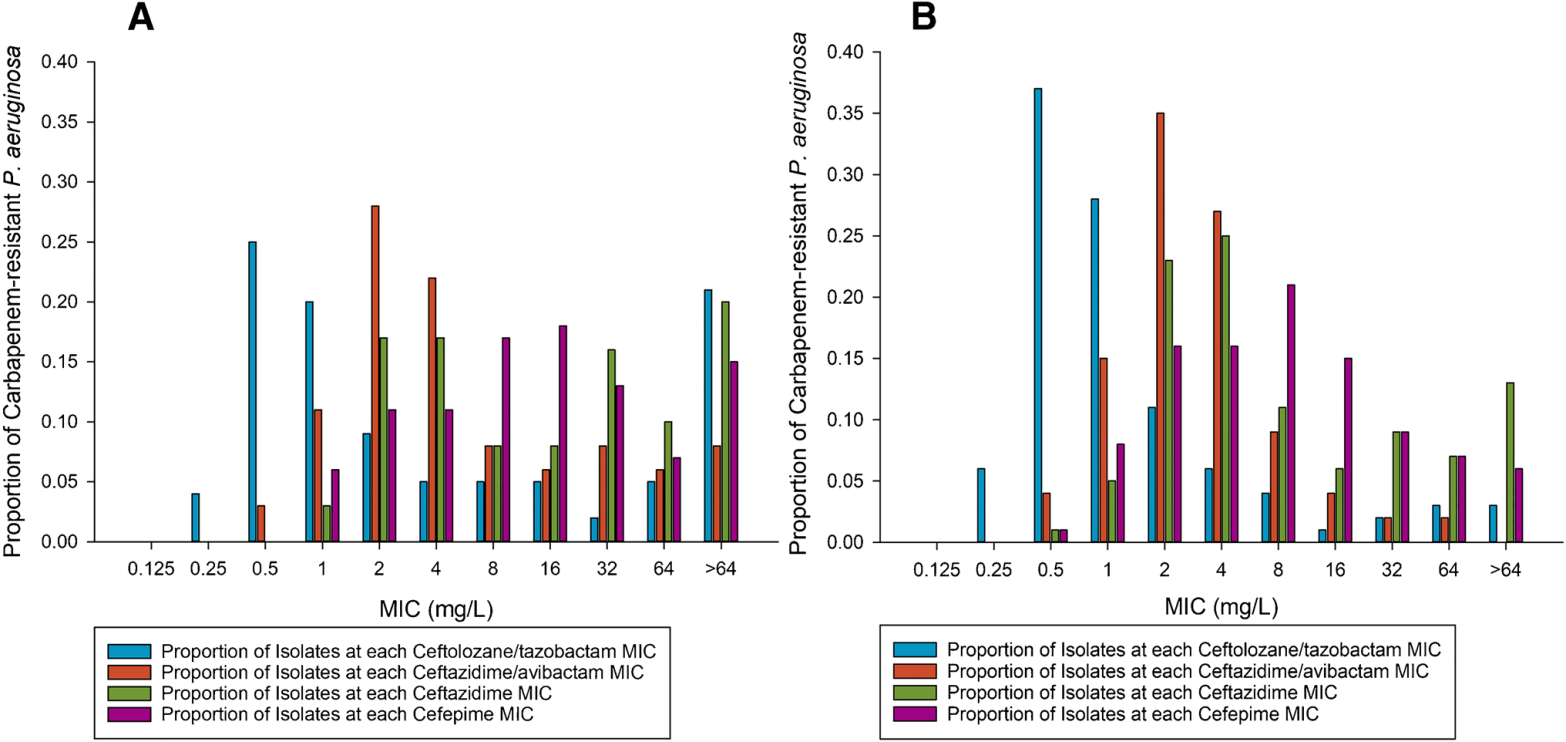
**a** MIC distribution of tested agents in the entire cohort. Ceftolozane/tazobactam: MIC_50/90_ 2/ > 64 mg/L, 63% susceptible; Ceftazidime/avibactam: MIC_50/90_ 4/64 mg/L, 72% susceptible. Ceftazidime: MIC_50/90_ 16/ > 64 mg/L, 46% susceptible; cefepime MIC_50/90_ 16/ > 64, 46% susceptible. **b**. MIC distribution of tested agents in the phenotypically carbapenemase negative isolates. Ceftolozane/tazobactam: MIC_50/90_ 1/8 mg/L, 88% susceptible; Ceftazidime/avibactam: MIC_50/90_ 2/8 mg/L, 91% susceptible. Ceftazidime: MIC_50/90_ 4/ > 64 mg/L, 65% susceptible; cefepime MIC_50/90_ 8/64, 63% susceptible

Against this global collection of carbapenem-resistant-*P. aeruginosa*, ceftolozane/tazobactam and ceftazidime/avibactam had MIC_50_/MIC_90_ values of 2/ > 64 mg/L and 4/64 mg/L, respectively. Ceftazidime/avibactam was the most active agent with 72% susceptibility per CLSI and EUCAST criteria followed by ceftolozane/tazobactam with 63% in all isolates. Both ceftazidime and cefepime remained susceptible against 46% of the carbapenem-resistant *P. aeruginosa*. Assessing isolates that tested phenotypically negative for carbapenemase production, more isolates tested susceptible to ceftolozane/tazobactam and ceftazidime/avibactam with 88 and 91% susceptibility, respectively. The phenotypic profiling of all isolates is presented in Fig. 1a, and the MIC distribution specific to phenotypically carbapenemase negative isolates is presented in Fig. 1b. Of note, a high proportion of serine-carbapenemase harboring isolates (KPC, *n* = 8; GES, *n* = 59) tested ceftazidime/avibactam susceptible with MIC_50_/MIC_90_ values of 4/8 and 2/8 mg/L, respectively. Table 3 displays the susceptibility testing results by each carbapenemase class.

**Table 3:** Antimicrobial susceptibility testing results of ceftolozane/tazobactam, ceftazidime/avibactam, and comparator anti-pseudomonal cephalosporins in carbapenem-resistant *P. aeruginosa* from the ERACE-PA Global Study Program by carbapenemase class identified

Subgroup	Antimicrobial susceptibility testing results						
	MIC (mg/L)		CLSI			EUCAST	
	MIC_50_	MIC_90_	%S	%I	%R	%S	%R
VIM, *n* = 136							
Ceftolozane/tazobactam	> 64	> 64	1%	0%	99%	1%	99%
Ceftazidime/avibactam	32	> 64	4%	–	96%	4%	96%
Ceftazidime	64	> 64	2%	12%	86%	2%	98%
Cefepime	32	> 64	8%	27%	65%	8%	92%
GES, *n* = 59							
Ceftolozane/tazobactam	16	64	2%	30%	68%	2%	98%
Ceftazidime/avibactam	2	8	90%	–	10%	90%	10%
Ceftazidime	32	> 64	2%	25%	73%	2%	98%
Cefepime	16	64	29%	37%	34%	29%	71%
IMP, *n* = 15							
Ceftolozane/tazobactam	> 64	> 64	0%	0%	100%	0%	100%
Ceftazidime/avibactam	> 64	> 64	0%	–	100%	0%	100%
Ceftazidime	> 64	> 64	0%	0%	100%	0%	100%
Cefepime	> 64	> 64	0%	0%	100%	0%	100%
NDM, *n* = 13							
Ceftolozane/tazobactam	> 64	> 64	0%	0%	100%	0%	100%
Ceftazidime/avibactam	> 64	> 64	0%	–	100%	0%	100%
Ceftazidime	> 64	> 64	0%	0%	100%	0%	100%
Cefepime	> 64	> 64	0%	0%	100%	0%	100%
KPC, *n* = 8							
Ceftolozane/tazobactam	> 64	> 64	12.5%	12.5%	75%	12.5%	87.5%
Ceftazidime/avibactam	4	8	100%	–	0%	100%	0%
Ceftazidime	> 64	> 64	12.5%	12.5%	75%	12.5%	87.5%
Cefepime	> 64	> 64	12.5%	12.5%	75%	12.5%	87.5%

The MIC results by region are presented in Table 4. Regional differences in susceptibility patterns were noted with ceftolozane/tazobactam susceptibility ranged from 32 to 85%. Similarly, ceftazidime/avibactam susceptibility ranged from 34 to 87%. For comparison, similar ranges were observed with ceftazidime and cefepime with susceptibility ranges of 22 to 56% and 14 to 60%, respectively.

**Table 4:** Antimicrobial susceptibility testing results of ceftolozane/tazobactam, ceftazidime/avibactam and comparator anti-pseudomonal cephalosporins in carbapenem-resistant *P. aeruginosa* from the ERACE-PA Global Study Program (*n* = 807)

Subgroup	Antimicrobial susceptibility testing results						
	MIC (mg/L)		CLSI			EUCAST	
	MIC_50_	MIC_90_	%S	%I	%R	%S	%R
Europe, *n* = 324							
Ceftolozane/tazobactam	1	> 64	65%	6%	29%	65%	35%
Ceftazidime/avibactam	4	32	79%	–	21%	79%	21%
Ceftazidime	8	> 64	52%	8%	40%	52%	48%
Cefepime	16	64	46%	24%	30%	46%	54%
Middle East, *n* = 163							
Ceftolozane/tazobactam	8	> 64	47%	7%	46%	47%	53%
Ceftazidime/avibactam	4	> 64	57%	–	43%	57%	43%
Ceftazidime	32	> 64	33%	8%	59%	33%	67%
Cefepime	16	> 64	42%	9%	49%	42%	58%
United States, *n* = 149							
Ceftolozane/tazobactam	1	16	85%	4%	11%	85%	15%
Ceftazidime/avibactam	2	16	87%	–	13%	87%	13%
Ceftazidime	8	> 64	56%	7%	37%	56%	44%
Cefepime	8	64	60%	20%	20%	60%	40%
South America, *n* = 106							
Ceftolozane/tazobactam	1	> 64	66%	2%	32%	66%	34%
Ceftazidime/avibactam	4	32	75%	–	25%	75%	25%
Ceftazidime	8	> 64	51%	8%	41%	51%	49%
Cefepime	8	> 64	50%	17%	33%	50%	50%
Africa, *n* = 65							
Ceftolozane/tazobactam	> 64	> 64	32%	0%	68%	32%	68%
Ceftazidime/avibactam	32	> 64	34%	–	66%	34%	66%
Ceftazidime	32	> 64	22%	3%	75%	22%	78%
Cefepime	32	> 64	14%	14%	72%	14%	86%

## Discussion

In a global collection of carbapenem-resistant *P. aeruginosa*, 33% of isolates tested phenotypically positive for carbapenemase production which varied based on region. Considering this high prevalence of carbapenemases, ceftolozane/tazobactam and ceftazidime/avibactam remained highly active against this collection of carbapenem-resistant *P. aeruginosa* five years into their use. Ceftazidime/avibactam remained highly active against the identified serine-carbapenemase producing isolates, further highlighting the importance of β-lactamase identification to guide therapy in the clinic.

Similar to previously assessed cohorts, VIM was the most commonly encountered carbapenemase in our study [18]. Notably detection of GES was the second most commonly identified in this cohort and is a growing clinical concern [19]. Detection of GES was most common in Europe; however GES harboring isolates were also identified in the Middle East and Africa. Although none of the US collected isolates in the present study tested positive for GES, recent reports have described their occurrence in the USA [20, 21]. These data call for introduction of commercially available assays that detect GES to better identify and subsequently help clinicians ascertain the most likely active antimicrobials against GES-harboring *P. aeruginosa*. IMP-harboring *P. aeruginosa* have been considered endemic to South East Asia [22]. The present study identified IMP harboring isolates from both the Middle East and South America further confirming global spread. A strength of the present study was the systematic approach where all isolates underwent phenotypic carbapenemase screening prior to genotypic assessment (CarbaR, CarbaR NxG, and WGS) considering that some carbapenemases may be outside the spectrum of current genotypic assays [14, 23]. Previous reports have shown that mCIM testing has excellent sensitivity (i.e., 98%) and would capture isolates outside of the scope of commercially available genotypic testing platforms (i.e., SPM and some IMP) [14, 15, 23]. However, false negatives are possible particularly among subtypes with poor hydrolytic activity (e.g., GES) [15, 23]. Additionally, with further implementation of carbapenemase-detection for carbapenem-resistant *P. aeruginosa* into clinical practice, periodic assessments on a local and global level should be conducted to detect shifts in carbapenemase prevalence and diversity to dictate local best practices for empiric therapy.

Previous data have supported the in vitro potency of ceftolozane/tazobactam and ceftazidime/avibactam against carbapenem-resistant *P. aeruginosa*. Indeed, susceptibility to both agents was highest in the USA consistent with a multicenter assessment that previously found 91 and 81% of isolate testing susceptible to each agent, respectively [5]. This high proportion of isolate testing susceptible to ceftolozane/tazobactam and ceftazidime/avibactam is likely secondary to the prominence of porin alterations and cephalosporinase over-production driving carbapenem-resistance. Considering the higher prevalence of carbapenemases globally, an assessment of meropenem-non-susceptible isolates from 2012 to 2014 found 72% susceptibility to ceftazidime/avibactam similar to the 72% susceptibility presented here [24]. Specific to an assessment of European and South American countries, ceftolozane/tazobactam remained active against 65% of carbapenem-non-susceptible *P. aeruginosa* in both regions compared with 65% and 66% of carbapenem-resistant isolates in the present study, respectively [25, 26]. The lowest ceftolozane/tazobactam and ceftazidime/avibactam susceptibility was observed in the Middle East/African sites. This is consistent with the high prevalence of metallo-β-lactamases observed in the present study and previous assessments from other countries in the region [27–29]. Assessments of novel agents or combinations with activity against both serine- and metallo-β-lactamase-producing *P. aeruginosa* are urgently needed in areas with such high prevalence of isolates harboring each or both enzyme classes.

Another underappreciated observation of the present study was that nearly 60% of carbapenem-resistant *P. aeruginosa* were isolated outside the ICU. While these findings are not ne [30], they have a tremendous impact on appropriate empiric therapy for the non-ICU patient population. These data further appeal for clinicians to consider early therapy that is active against carbapenem-resistant *P. aeruginosa* as part of empiric therapy guidelines outside of the intensive care units. The use of rapid molecular diagnostics will also help guide therapeutic decisions both within and outside the ICU.

The present study is not without limitations. Indeed, whole genome sequencing was not conducted for all carbapenemase positive isolates, so individual carbapenemase alleles were outside of the scope of the present study. However, we had a rigorous assessment for genotypic carbapenemases detection that included the commercially available CarbaR and the CarbaR NxG provides an expanding insight into the molecular detection of carbapenemases outside of only the “Big Five.” Additionally, this approach has translational benefit since healthcare providers in the clinical setting are increasingly making therapeutic decisions based on commercially available genotypic assays. Similarly, mutations in chromosomal resistance mechanisms have been described to dictate ceftolozane/tazobactam and ceftazidime/avibactam susceptibility[11] however based on the molecular methods used were not assessed here.

In conclusion, the findings of the present study re-affirm the potency of ceftolozane/tazobactam and ceftazidime/avibactam against a global collection of carbapenem-resistant *P. aeruginosa* 5 years into marketing. Clinicians should consider the local prevalence and diversity of carbapenemases among *P. aeruginosa* to guide antimicrobial therapy as their presence may dramatically change the ceftolozane/tazobactam and ceftazidime/avibactam susceptibility profile. Rapid carbapenemase-detection may help direct empiric therapy to ceftolozane/tazobactam, ceftazidime/avibactam, or alternative agents sooner in the clinical course prior to conventional susceptibility testing results. Additionally, the ERACE-PA Global Surveillance Program provides a contemporary collection of carbapenem-resistant *P. aeruginosa* to study therapeutic optimization for this challenging pathogen.

## Data Availability

Data are available through inquiry with the corresponding investigator.
